# Site-controlled telecom-wavelength single-photon emitters in atomically-thin MoTe_2_

**DOI:** 10.1038/s41467-021-27033-w

**Published:** 2021-11-19

**Authors:** Huan Zhao, Michael T. Pettes, Yu Zheng, Han Htoon

**Affiliations:** Center for Integrated Nanotechnologies, Materials Physics and Applications Division, Los Alamos National Laboratory, Los Alamos, New Mexico 87545 USA

**Keywords:** Two-dimensional materials, Quantum optics, Single photons and quantum effects

## Abstract

Quantum emitters (QEs) in two-dimensional transition metal dichalcogenides (2D TMDCs) have advanced to the forefront of quantum communication and transduction research. To date, QEs capable of operating in O-C telecommunication bands have not been demonstrated in TMDCs. Here we report site-controlled creation of telecom QEs emitting over the 1080 to 1550 nm telecommunication wavelength range via coupling of 2D molybdenum ditelluride (MoTe_2_) to strain inducing nano-pillar arrays. Hanbury Brown and Twiss experiments conducted at 10 K reveal clear photon antibunching with 90% single-photon purity. The photon antibunching can be observed up to liquid nitrogen temperature (77 K). Polarization analysis further reveals that while some QEs display cross-linearly polarized doublets with ~1 meV splitting resulting from the strain induced anisotropic exchange interaction, valley degeneracy is preserved in other QEs. Valley Zeeman splitting as well as restoring of valley symmetry in cross-polarized doublets are observed under 8 T magnetic field.

## Abstract (web summary)

Single-photon emitters in 2D semiconductors hold promise for quantum applications, but usually operate in the 500-800 nm wavelength range. Here, the authors report site-controlled creation of quantum emitters in the telecommunication wavelength window by coupling 2D MoTe2 to strain inducing nano-pillar arrays.

## Introduction

Quantum emitters (QEs) that emit one photon at a time are key building blocks for numerous quantum technology protocols such as quantum communications, quantum information processing, and quantum key distribution^1–4^. In particular, QEs operating in the telecom bands (1.25–1.55 μm) are highly desired for implementation of quantum technologies through existing fiber-based optical communication networks. Currently, telecom-compatible QEs have been demonstrated in various III–V semiconductor quantum dots^5,6^ and recently in functionalized carbon nanotubes^7^. However, several challenges such as accurate site positioning and efficient polarization control still remain for these QEs. Over the past decade, two-dimensional (2D) semiconductors have emerged as a novel platform for both fundamental research and technological applications. Leveraged by the unique membrane-like geometry, 2D semiconductors are promising for QEs, as they offer high photon extraction efficiency, easy coupling to external fields, and convenient integration with photonic circuits. In addition, strain engineering can be applied to accurately position the emission sites^8–11^. Most importantly, 2D transition metal dichalcogenides (TMDCs) have a valley degree of freedom that can be manipulated and accessed through circularly polarized excitonic optical transitions and efficiently tuned via a magnetic field^12,13^, bringing new quantum functionalities to embedded QEs. Recently, 2D QEs have been demonstrated in WSe_2_^14–17^ and hexagonal boron nitride^18,19^, covering a broad emission spectrum range from ~500 to 800 nm. However, single-photon emission in the most desirable spectral range—the telecom bands—has never been explored in 2D systems. Although most 2D semiconductors have inherent electronic band structures that limit the operating wavelength to the visible range, *α*-phase 2H-molybdenum ditelluride (MoTe_2_) has a layer-dependent bandgap in the NIR regime, holding promise for telecom-compatible single-photon emission. However, extending the nanoscale strain engineering approach to MoTe_2_ has been a major technical challenge. Although QEs can even form naturally^14–17^ and can be induced with almost any type of nanoscale topological features starting from bubbles^20^ to lithographically defined patterns^8–11^ in WSe_2_, no natural QEs have been reported in MoTe_2_ and whether MoTe_2_ could host QEs remains unexplored.

Here we report site-controlled telecom single-photon emission in MoTe_2_ mono- and few layers by transferring mechanically exfoliated MoTe_2_ thin flakes onto strain-inducing nano-pillar arrays. We observed QEs in both monolayer and relatively thick (>10 layers) MoTe_2_ flakes, with emission wavelength covering the entire near-infrared (NIR) emission band from 1080 nm to 1550 nm. Our Hanbury Brown and Twiss (HBT) experiment yielded near-complete photon antibunching, unambiguously proving the single-photon nature of the emitters. Polarization-resolved magneto-optical spectroscopies further revealed that although valley symmetry is preserved in some QEs, the strain-induced anisotropic exchange interaction can mix valley states in other QEs to display cross-linearly polarized doublets with ~1 meV splitting at zero magnetic field. The valley symmetry in these QEs is restored under 8 T magnetic field.

## Results

### Near-band-edge QEs in monolayer MoTe_2_

**Fig. 1:**
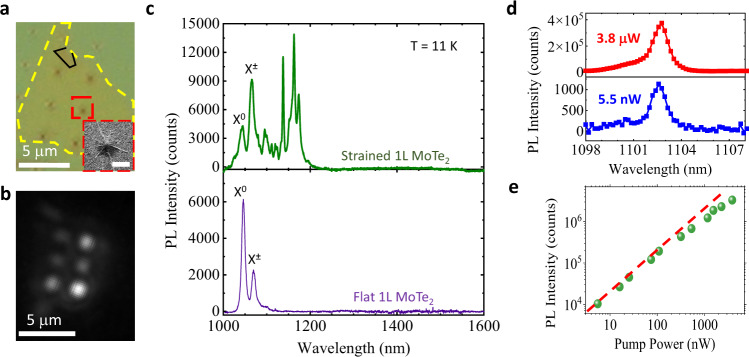
Near-band-edge sharp emission lines obtained from strained monolayer MoTe_2_. **a** Optical image of a monolayer MoTe_2_ flake (with a tiny folded bilayer region outlined in black) on a nano-pillar array. The MoTe_2_ flake is outlined by yellow dashed lines. Scale bar: 5 μm. Inset: SEM image of a nano-pillar coated with monolayer MoTe_2_, displaying a tent-shaped profile. Scale bar of the inset: 500 nm. **b** Wide-field PL image of the same flake. The brighter areas indicate stronger emission and are consistent with the pillar locations in Fig. 1a. Scale bar: 5 μm. **c** PL spectrum of a MoTe_2_ monolayer on flat PMMA (lower panel) and the PL spectrum of a MoTe_2_ monolayer sitting on a nano-pillar (upper panel) acquired at laser excitation power of 0.8 μW and 30 s integration time. The MoTe_2_ exciton and trion peaks are identified. **d** PL spectra of a representative localized MoTe_2_ emitter acquired at 3.3 μW and 5.5 nW pump-power showing invariant shape and width (920 µeV at FWHM) of the spectral line. **e** Pump-power-dependent emission intensity of the PL peak presented in Fig. 1d shows near-perfect linear scaling with pump-power. The red dashed line is a guide to the eye. All data were obtained at 11 K temperature.

Figure 1a presents an optical image of a MoTe_2_ monolayer on a nano-pillar array. The sample preparation and nanofabrication processes are discussed in “Methods” section and Supplementary Note 1. The corresponding wide-field photoluminescence (PL) image (Fig. 1b) shows significantly brighter PL emission in the regions directly contacting the nano-pillars. Our initial efforts using naturally formed bubbles and lithographically defined Si nanostructures to induce strain in MoTe_2_ only produced broad featureless emissions (Supplementary Fig. 3). Unstrained monolayer MoTe_2_ features two dominant emission peaks at cryogenic temperature: exciton (*X*^0^) emission at around 1050 nm and trion (*X*^±^) emission at around 1070 nm (Fig. 1c, lower panel)^21,22^. With localized strain, a series of narrow PL peaks covering a broad spectral range of 1080–1150 nm emerges from the lower-energy side of the spectrum (Fig. 1c upper panel and Supplementary Fig. 4). A single nano-pillar often produces multiple sharp PL peaks at slightly different spatial locations (determined through the centroid of the PL emission). Following recent theoretical studies, we attribute these PL peaks to the localized exciton states arising from strain-induced hybridization of dark excitons and the defect states^23,24^. As our nano-pillars induce deformation over several hundreds of nanometers (Supplementary Fig. 2) and are thus capable of hosting multiple point defects, spatially distinct localized exciton states could form in a single strained region. An increase in PL intensity of trion (*X*^±^) emission relative to that of *X*^0^ emission in the strained region further indicates that the redistribution of free carriers under non-uniform strain profiles observed previously in WS_2_^25^ could also occur in our case and localized exciton states could also be charged. The linewidths of the QE PL emission range from a few meV to sub-meV at 4–10 K temperatures, which are nearly an order of magnitude narrower than the linewidths of the 2D exciton PL peaks (~10 meV). Supplementary Fig. 5 displays a narrower PL peak of 0.6 meV full width at half maximum (FWHM) observed at 4 K. These narrow, near-band-edge emission lines are also frequently observed in two to four layered MoTe_2_ but are not seen in much thicker flakes. Figure 1d shows a typical narrow PL peak from a QE that displays a FWHM of 920 μeV. The peak is accompanied by a weak shoulder peak at ~2.5 meV higher energy. Both the width and shape of this PL peak remain essentially unchanged over nearly three orders of magnitude change in pump-power. The PL intensity also varied linearly with pump-power and only showed weak saturation at powers >300 nW (Fig. 1e).

**Fig. 2:**
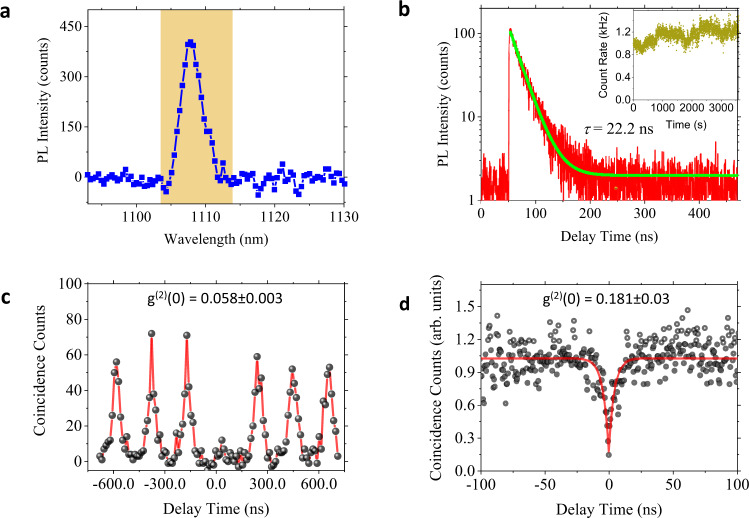
PL dynamics of a near-band-edge MoTe2 QE. **a** PL spectrum of a localized MoTe_2_ emitter. The data of Fig. 2b–d are taken from this QE with a band-pass filter that allows the shadowed region to be detected. **b** The PL decay curve (red) and a single-exponential decay fit (green) with a 22.2 ± 0.1 ns extracted lifetime. Inset: PL intensity as a function of experiment time showing stability of the single-photon emission rate over 1 h time, the modulation observed over the long timescale (~100 s) is mainly due to sample drift relative to the laser excitation focal spot. **c** Second-order correlation measurement under 850 nm pulsed excitation with a 2.1 MHz repetition rate, from which a *g*^(2)^(0) = 0.058 ± 0.003 is extracted. **d** Second-order correlation measurement under 850 nm CW laser excitation. The red curve is a fit to the data using a bi-exponential decay function. The extracted *g*^(2)^(0) of 0.181 ± 0.030 is slightly higher than the pulsed measurement due to higher Poissonian background caused by CW excitation. All data were obtained at 11 K temperature.

To access the single-photon-emission characteristics of the QEs, we performed time-tagged, time-correlated single-photon counting and HBT experiments. The PL intensity time trace, PL decay curve, and second-order correlation at zero time delay, or *g*^2^(0), was extracted from the photon stream. Figure 2a displays the PL spectrum of a localized emitter, of which the measurement results are presented in Fig. 2b–d. The PL decay curve (Fig. 2b) shows a near-perfect single-exponential decay with a lifetime of *τ* = 22.2 ± 0.1 ns, four orders of magnitude longer than that in pristine MoTe_2_ (~2 ps)^26^. This long lifetime provides a clear indication that localization of the exciton in a strain-induced potential trap suppresses non-radiative recombination through defect states that dominate the decay of 2D band-edge excitons.

Quantum-dot-like solid-state QEs typically have instability issues such as photobleaching, blinking, and spectral diffusion—all of which hinder applications^27^. We monitored the time-dependent PL emission under both pulses and continuous wave (CW) laser excitation (Fig. 2b inset and Supplementary Fig. 6), revealing stable emission at an average count rate of 1.2 and 9 kHz for pulsed and CW excitation, respectively, without detectable photobleaching or blinking over the timescales presented. A time series of PL spectra with 0.4 meV spectral resolution also show no spectral diffusion (Supplementary Fig. 7). Figure 2c, d present the photon correlation under pulsed [*g*^(2)^(0) = 0.058 ± 0.003] and CW [*g*^(2)^(0) = 0.181 ± 0.030] excitation, respectively. Both values are well below the photon antibunching threshold of *g*^(2)^(0) = 0.5, which unequivocally reveals that the strain-induced MoTe_2_ localized emitter is indeed a QE. HBT experiments conducted on a different emitter at 40 and 77 K prove that the single-photon-emission behavior can survive up to liquid nitrogen temperature, although the emission intensity decreases significantly due to thermally activated non-radiative recombination (Supplementary Fig. 8). Normalizing the measured count rates of the QE of Fig. 2 with the overall collection efficiency of our micro-PL system at 1108 nm (0.035%, see Supplementary Note 4), we estimate the corrected emission rate of the QE to be 3.4 and 25.6 MHz for pulsed and CW excitation, respectively. Assuming that an exciton is localized into the QE in every excitation cycle, we can further deduce the radiative recombination efficiency of the QE to be ~7% from the ratio between the corrected emission rate under pulsed excitation and the 48.5 MHz pulsed excitation rate^8,28,29^.

### Telecom-wavelength QEs in multilayer MoTe_2_

**Fig. 3:**
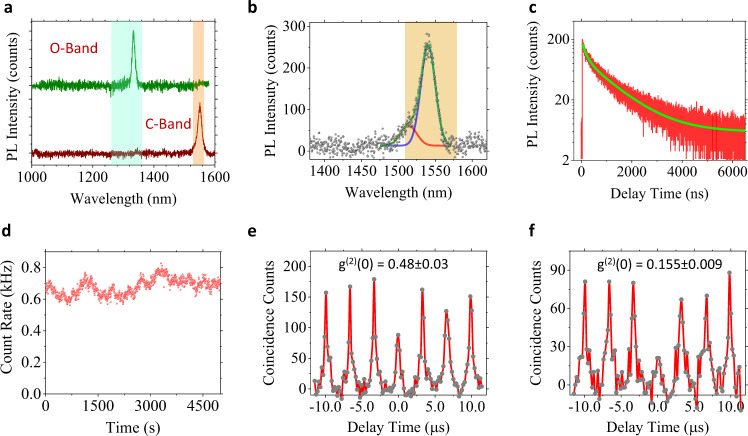
PL spectra and PL dynamics of telecom QEs. **a** “O-band” and “C-band” telecom PL emissions from two multilayer MoTe_2_ localized emitters, corresponding telecom bands indicated by shaded regions. **b** MoTe_2_ QE with a 1540 nm telecom emission peak and a small shoulder peak at 1510 nm. The blue and red lines are Gaussian fits to the 1540 and 1510 nm peaks, respectively. The data of Fig. 3c–f are taken from this emitter with a band-pass filter that allows only the shaded region to be detected. **c** PL decay curve (red) and bi-exponential decay fit (green) reveal the lifetime of the 1540 nm peak is 1.13 ± 0.01 μs. **d** Time-dependent PL counts showing stable emission over 5000 s. Data are recorded every 500 ms. **e** Second-order correlation measurement under 850 nm pulsed excitation with a 330 kHz repetition rate, from which *g*^(2)^(0) = 0.48 ± 0.03 is extracted. **f** Time-gated *g*^(2)^ shows *g*^(2)^(0) = 0.155 ± 0.009 under a 200 ns time gate. All data were obtained at 13 K temperature.

Obtaining telecom-compatible QEs that emit at around 1.3 μm (O-band, “original band”) and 1.55 μm (C-band, “conventional band”) are required for fiber-based quantum communications. When increasing the MoTe_2_ layer numbers from monolayer to bulk, the bandgap of MoTe_2_ monotonically decreases from 1.18 eV (1050 nm) to 0.95 eV (1300 nm)^22^. The PL intensity also decreases by orders of magnitude due to a direct-to-indirect bandgap transition commonly observed in 2D TMDCs. In our experiment, we observe bright telecom-band emission created from strained few-layer MoTe_2_, although we have occasionally found such emissions in mono- and bilayer samples (Supplementary Notes 7–9). Figure 3a and Supplementary Fig. 9 present PL spectra of telecom-wavelength emitters. We typically observe such highly red-shifted bright emissions spanning 1.25–1.55 μm, covering the full telecom window. Supplementary Fig. 10 demonstrates that QEs emitting at wavelengths longer than 1300 nm are created in five- to six-layer MoTe_2_ at almost all nano-pillar sites. In contrast to near-band-edge QEs, these QEs are characterized by relatively broad linewidths (FWHM 7 ~ 30 meV).

Figure 3b presents the PL spectrum of a telecom QE, for which the PL dynamics and photon correlation results are presented in Fig. 3c–f. We observed an initial PL decay with a lifetime of 163 ± 3 ns, followed by an ultra-long lifetime of 1.13 ± 0.01 μs. This ultra-long lifetime is attributed to the dominant PL peak at lower energy, confirmed by comparing the integrated PL counts from the PL decay curve with the PL counts in the spectrum (see Supplementary Note 10). The measured lifetime is six orders of magnitude longer than that of the MoTe_2_ band-edge emission and is two to three orders longer than that of our near-band-edge QEs. Based on this long lifetime and the fact that these telecom QEs are observed more frequently in multilayer MoTe_2_, we attribute the telecom QEs to indirect excitonic transitions, which are activated by strain-induced quantum confinement potentials. Figure 3d shows that this telecom QE is also free from blinking or photobleaching over a 5000 s time period. The HBT experiment under pulsed excitation for the spectral window shown in Fig. 3b yields *g*^(2)^(0) = 0.48 ± 0.03 (Fig. 3e). As this measurement includes contribution from a high-energy shoulder that exhibits a shorter PL decay time constant, we employed a time-gated *g*^(2)^ experiment^30^ (Supplementary Note 11), in which only the photons arriving after the decay of the higher energy shoulder (i.e., after 200 ns delay) were analyzed for the *g*^(2)^ trace. The time-gated *g*^(2)^ in Fig. 3f shows *g*^(2)^(0) = 0.155 ± 0.009, clearly proving the antibunching nature of the 1540 nm telecom emitter. HBT experiments of two other QEs emitting at 1340 (O-band) and 1233 nm yielded *g*^(2)^(0) of 0.28 ± 0.02 and 0.10 ± 0.01, respectively, and are shown in Supplementary Fig. 11. Using the overall collection efficiency of our micro-PL system at 1550 nm (0.31%) (Supplementary Note 4) and 303 kHz pulsed excitation rate, we can estimate the absolute emission rate and radiative recombination efficiency of the QE shown in Fig. 3 to be 258 kHz and 85%, respectively^8,28,29^.

### Polarization-resolved magneto-optical spectroscopies

**Fig. 4:**
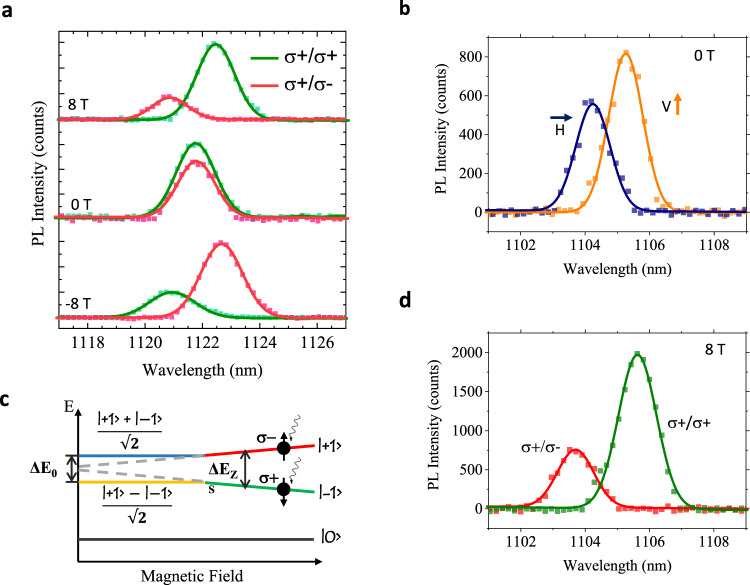
Polarization-resolved magneto-optical spectroscopies. **a** Helicity-resolved magneto-PL data of a MoTe_2_ QE. The emitter was excited using a *σ*^+^ polarized laser. **b** The PL spectrum of a linearly cross-polarized doublet measured at zero field, showing a fine-structure energy splitting of ~1.1 meV. H and V denote horizontal and vertical polarization detection directions, respectively. **c** Energy diagram of the doublet as a function of the external magnetic field. The linearly polarized states (yellow and blue lines) are converted into *σ*^+^ and *σ*^−^ circularly polarized states (green and red lines) once the Zeeman energy (Δ*E*_Z_) exceeds the zero-field fine-structure splitting energy (Δ*E*_0_). The Zeeman splitting energy at linear polarization state is indicated by the gray dash lines. The ground state is denoted as |0〉. All data were obtained at 11 K temperature. **d** Spectrum of the same doublet under an 8 T magnetic field, showing the doublet is converted to a circularly cross-polarized pair. The excitation is a *σ*^+^ polarized laser. Lines in Fig. 4a–c are Gaussian peak fits to the PL data.

To investigate the valley physics of QEs in MoTe_2_, we conducted polarization-resolved magneto-PL spectroscopy with magnetic field normal to the sample surface (Faraday geometry). Figure 4a presents helicity-resolved PL of a MoTe_2_ QE. The spectra was taken with *σ*^+^ excitation and analyzed for both *σ*^+^ and *σ*^−^ helicities. The valley Zeeman splitting was not observed in the absence of a magnetic field but rose with increasing field, indicating a lifting of valley degeneracy. Using the relation between the energy splitting Δ*E* and the magnetic field **B**, Δ*E* = − *gμ*_B_**B**, where *μ*_B_ is the Bohr magneton, we extracted a Landé *g*-factor of −3.61 ± 0.02 for the QE (Supplementary Note 12), which is comparable with *g*-factors reported in other work^31^. At zero field, the degree of circular polarization, *P*_C_ = (*I*_*σ*+_ − *I*_*σ*−_)/(*I*_*σ*+_ + *I*_*σ*−_) was 13% as a result of the valley-selective pumping effect. Significant valley polarization was observed with an applied magnetic field, reaching 52% at 8 T, which is more pronounced compared to the 30% polarization reported in magnetic-field-induced valley polarization of intrinsic MoTe_2_ excitons^31^. These results further indicate a vanishing fine-structure splitting of some of the QEs at zero field that could arise from azimuthally symmetric strain-induced confinement potentials^32^. In this case, these QEs hold great potential for entangled photon generation^33^. However, vanishing fine-structure splitting could also result from charging of the QE^34,35^. As the g-factor of neutral and charged excitons of MoTe_2_ are almost identical (−4.6 ± 0.2 for exciton and −4.5 ± 0.3 for trion)^31^, our magneto-PL experiment cannot be used to distinguish these two possibilities, similar to the case of WSe_2_ QEs^16,32,34,35^.

We also observed emission pairs in some of the QEs, which were found to be cross-linearly polarized doublets with sizeable zero-field energy splitting (1–3.7 meV) (Fig. 4b and Supplementary Note 13). The two peaks from the doublet have a zero-field splitting of 1.09 meV and reach their maximal PL intensities in opposite linear polarization directions (horizontal/vertical), indicating the presence of fine structure. The observed cross-polarization and zero-field energy splitting have also been reported in III–V quantum dots^36,37^ and recently in WSe_2_ QEs^15,17^. The higher PL intensity of the lower-energy state is attributed to the efficient thermal relaxation process that has also been reported in WSe_2_ QEs^15,17^. Following prior studies, we attribute this fine-structure splitting to hybridization of K and K’ valley-polarized excitons by an asymmetric potential landscape defined by the localized strain as illustrated in Fig. 4c. When the magnetic field is strong enough to overcome the anisotropic Coulomb potential (i.e., Δ*E*_Z_ > Δ*E*_0_), the linearly polarized states vanish and circularly polarized states are recovered. This restoration of valley symmetry is achieved in our QE under an 8 T magnetic field (Fig. 4d). Compared to the ~0.7 meV fine-structure splitting energy of as-exfoliated WSe_2_ QEs^15,17^, the larger zero-field splitting energy in our MoTe_2_ QEs suggests that the nano-pillar-based strain engineering technique can induce more confining potential anisotropy compared to native strain.

## Discussion

In this work, we report site-controlled creation of two types of QEs using nano-pillar-based strain engineering: near-band-edge QEs covering 1080–1150 nm in mono- and few-layer MoTe_2_ and telecom QEs emitting in 1200–1600 nm range in multilayer MoTe_2_. The quantum natures of the QEs were verified by photon correlation measurements up to liquid nitrogen temperature. Compared to telecom QEs, the near-band-edge QEs has a relatively low radiative recombination efficiency, which could result from thermal hopping of excitons out of the shallow trapping potential. This efficiency increases significantly when the QE is isolated in energy from the band-edge, as demonstrated in our telecom QEs. The deep-trapping potential of the telecom QEs strongly suppresses thermal de-trapping of excitons, which is believed to be responsible for the low radiative efficiency of band-edge QEs. These results indicate that we can utilize the thickness of the MoTe_2_ layer as a control knob for the creation of two types of QEs. In contrast, only near-band-edge QEs can be created in WSe_2_^8,14–17,34,35,38^ and only deep (110–220 meV) trap states with long lifetime can be created in MoS_2_^39^. Although the long PL lifetime and broad emission linewidth of telecom QEs are generally not desirable as they could limit generation rate and indistinguishability of single photons, recent studies have shown that both of these limitations can be overcome by exploiting plasmonic enhancement^8,40^. As in the case of all 2D QEs, control over the emission wavelength remains a major challenge. In addition, we observed cross-polarized doublets with ~1.1 meV zero-field energy splitting in some of the localized emitters, suggesting a strong anisotropic confinement. The polarization state of the QEs was found to be tunable by external magnetic field. Our findings extend the operating wavelength of 2D QEs into the NIR regime, bringing in solutions for creating site-controlled stable telecom-compatible QEs. We envisage various future directions inspired by our early-stage demonstration, including electrically driven telecom QEs and cavity-enhanced tunable NIR QEs. We are also encouraged by the possibility of realizing room-temperature MoTe_2_ telecom QEs, as the energy redshift between the telecom emission and the MoTe_2_ exciton emission is well above the thermal energy at room temperature. Finally, an in-depth understanding of the excitonic physics of MoTe_2_ QEs may give rise to interesting perspectives on manipulating the spin-valley degree of freedom and designing valleytronic devices.

## Methods

### Sample preparation

MoTe_2_ flakes were mechanically exfoliated from a flux-grown bulk crystal before they were transferred onto pre-patterned substrates. Thin layers (i.e., flakes that look greenish and translucent under an optical microscope) were selected for further optical characterizations. One- to four-layer-thick flakes can be easily distinguished by analyzing the band-edge emission wavelength and PL intensity^22^. Flakes thicker than four layers do not have a detectable band-edge emission using μW level pump-power. To prepare the strained substrates, a 50 nm Au layer was deposited on top of a Si/SiO_2_ substrate to block silicon PL emission, followed by spin-coating of a 50 nm polyvinyl alcohol (PVA) dielectric layer to prevent quenching effects. Then, a ~120 nm polymethyl methacrylate (PMMA) layer was spin-coated on top of the PVA layer and patterned by electron beam lithography into PMMA nano-pillar arrays with a 3 μm pitch width. Vacuum annealing at 90 °C was performed to enhance the contact between the 2D flakes and the nano-pillars. Each pillar had a ~100 nm pillar height and a ~150 nm diameter. It is worth noting that later we found the PVA spacer layer was not necessary as the ~100 nm PMMA pillars were sufficient to separate the MoTe_2_ dots from the gold layer and maintain efficient PL emission.

### Optical characterization

A diagram of our optical measurement setup is presented in Supplementary Note 14. Micro-PL measurements of MoTe_2_ QEs were performed on a home-built confocal microscope with excitation from either an 850 nm CW Ti:sapphire laser or an 850 nm supercontinuum pulsed laser. The excitation power was typically a few μW. Samples were mounted in a continuous flow cryostat and cooled to 10–13 K using liquid helium. The emitted light was collected through a ×50 infrared objective lens (Olympus, 0.65 NA) and spectrally filtered before entering a 2D InGaAs array detector (NIRvana 640LN, Princeton Instruments). We used 150 and 300 gr/mm gratings to resolve the spectra. For TRPL and HBT experiments, the emission signal was spectrally filtered before coupling into a 50 : 50 optical fiber beamsplitter, which equivalently split the signal into two beams and sent them into two channels of a superconducting nanowire single-photon detector (Quantum Opus). PL intensity time traces, PL decay curves, and g^(2)^ traces were obtained from photon detection events recorded by a PicoQuant HydraHarp 400 time-correlated single-photon-counting module. We applied a bi-exponential decay model to determine the CW *g*^(2)^(0) value and error level. For pulsed auto-correlation measurements, *g*^(2)^(0) was extracted by comparing the integrated photon coincidence counts at the zero time-delay peak with the averaged integrated photon coincidence counts at 30 adjacent peaks. The error level of pulsed *g*^(2)^(0) was defined by the SD of the integrated photon coincidence counts in the adjacent peaks. For the pulsed *g*^(2)^(0) measurement of the 1540 nm QE peak, a time gate of 200 ns was applied to significantly reduce the contribution from the undesired emissions that could not be fully removed by optical filters.

For magneto spectroscopy and polarization-resolved PL measurements, the sample was placed inside the room-temperature bore of an 8.5 T liquid-helium cooled superconducting magnet. For linear polarization analysis, the excitation beam was fully depolarized using a laser depolarizer to eliminate the polarization memory effect. A half-wave plate (HWP) was inserted into the collection channel to rotate the polarization direction. A Wollaston prism was placed between the HWP and the InGaAs detector to spatially split the emission into horizontal and vertical components, followed by a depolarizer to avoid effects arising from the linear polarization dependence of the gratings. For circular polarization analysis, a quarter-wave plate (QWP) was inserted into the shared path of the excitation and the emission beams. As a result, the QWP turns the linearly polarized laser into a circularly polarized excitation source and converts the circularly polarized PL signals into linearly polarized beams. A HWP and a Wollaston prism were installed in the collection beam to spatially split the *σ*^+^ and *σ*^−^ emissions into two different areas on the 2D InGaAs array.

## Supplementary information

Supplementary Information

## Data Availability

The data that support the findings of this study are available from the corresponding author upon request.
