# Supplementary material for: Site-controlled telecom-wavelength single-photon emitters in atomically-thin MoTe2
Source: Nat Commun. 2021 Nov 19;12:6753. doi: 10.1038/s41467-021-27033-w (PMC8604946; doi:10.1038/s41467-021-27033-w)
Supplement: Supplementary file 1 — Supplementary Information [file 41467_2021_27033_MOESM1_ESM.pdf]

Site-Controlled Telecom-Wavelength Single-Photon Emitters in Atomically-thin $MoTe_2$

Huan Zhao[1*], Michael T. Pettes[1], Yu Zheng, and Han Htoon[1*]

[1]Center for Integrated Nanotechnologies, Materials Physics and Applications Division, Los Alamos National Laboratory, Los Alamos, New Mexico 87545, USA

[*]Corresponding authors, Email: huanzha@lanl.gov, htoon@lanl.gov

- **Supplementary Note 1. Sample Preparation**
- **Supplementary Note 2: Spectra of 24 near-band-edge $MoTe_2$ localized emitters**
- **Supplementary Note 3: Comparison of QE PL linewidth at 14 K and 4.9 K**
- **Supplementary Note 4: Determining single photon detection efficiency and corrected emission rate of QEs**
- **Supplementary Note 5: Near-band-edge emitter working at liquid-nitrogen temperature**
- **Supplementary Note 6: PL time trace of a near-band-edge emitter**
- **Supplementary Note 7: Highly red-shifted localized emissions**
- **Supplementary Note 8: Nearly-unity emitter creation with high positioning accuracy**
- **Supplementary Note 9: Second-order correlation data of SPEs emitting from 1.1 to 1.6 μm.**
- **Supplementary Note 10: Correlating the PL spectra and TRPL decay curve of the QE shown in Figure 3b,c of the main article**
- **Supplementary Note 11: Photon correlation measurements and the time-gating technique**
- **Supplementary Note 12: Magnetic-field dependent valley Zeeman splitting**

- **Supplementary Note 13: A cross-linearly polarized doublet with an anomalously large zero field splitting**
- **Supplementary Note 14: A diagram of the optical measurement setup**

Supplementary Note 1. Sample Preparation

The nano-pillars were fabricated using the following procedures, see Supplementary Figure 1. Each nano-pillar has a diameter of ~150 nm and a height of ~100 nm. Supplementary Figure 2 demonstrates that after transferring thin-layer $MoTe_2$ and vacuum annealing at 90°C, strained structures were generated.

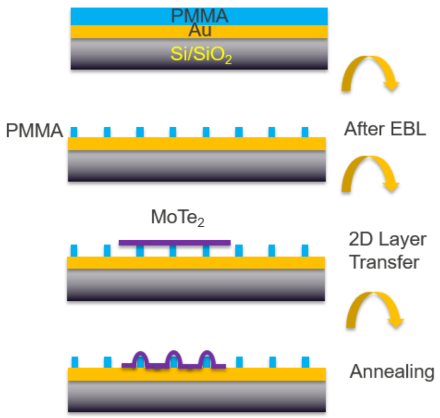

Supplementary Figure 1: The fabrication process for the $MoTe_2$-on-PMMA nano-pillar quantum emitters (QEs). PMMA nanopillars are fabricated using EBL. $MoTe_2$ flakes are transferred on top of the pillars with a dry transfer technique. The samples are annealed before measuring.

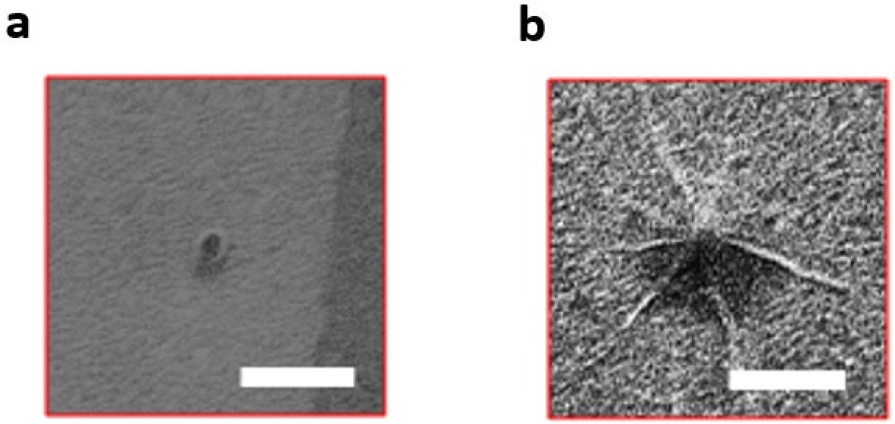

Supplementary Figure 2: SEM images of the nanopillar. Left panel: SEM image of one as-fabricated PMMA nano-pillar; Right panel: A nano-pillar coated by a $MoTe_2$ monolayer. Scale bars: 500 nm.

We also tested other strain engineering methods to modify the PL emission profiles of mono- and multilayer $MoTe_2$, which typically lead to very broad defect emission bands (Supplementary Figure 3). The strain engineering methods we tested including nano-bubbles, meta-material structures, and ultra-sharp dielectric tips. These “unsuccessful” strain-inducing media are either too flat to generate enough local strain, or too sharp/rough to host a monolayer $MoTe_2$ flake without breaking/piercing it.

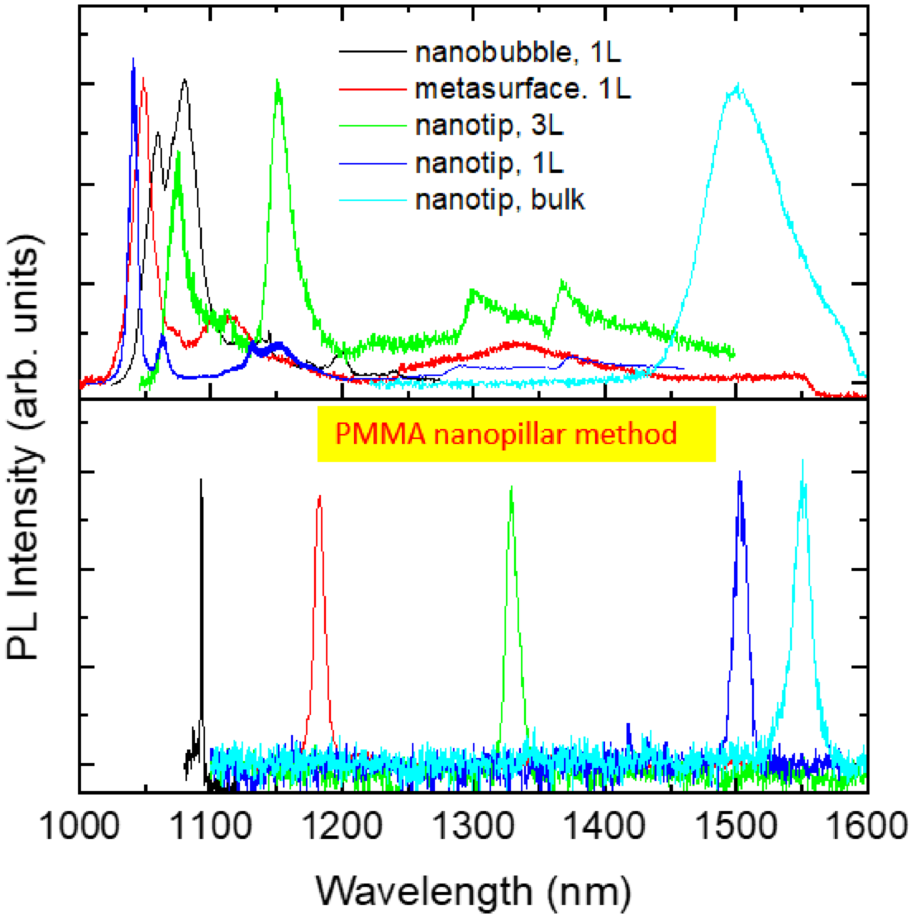

Supplementary Figure 3: A comparison of the $MoTe_2$ PL profile modulation through PMMA nanopillars (lower panel) with the PL profiles modulated by other strain-inducing methods we tested (upper panel). Our nanopillar method gives much better result.

Supplementary Note 2: Spectra of 24 near-band-edge $MoTe_2$ localized emitters

The near-band-edge emission PL lines are typically observed in ~1050–1070 nm (20-100 meV lower than $MoTe_2$ the bi-exciton) on <4-layer thick $MoTe_2$. They have significantly narrower linewidths compared to those of the largely redshifted emissions. Here we plot the PL emission of

24 strained few-layer $MoTe_2$ localized emitters. We only display the 1080–1150 nm wavelength range to show the linewidth clearly. All the spectra were taken at a temperature of 10–13 K. Multiple sharp PL peaks are often observed at slightly different spatial locations around a single nano-pillar (determined through the centroid of the PL emission). As our nano-pillars induce deformation over a region several hundreds of nm in diameter (see Supplementary Figure 2) capable of hosting multiple point defects, distinct localized exciton states can form inside a single strain region.

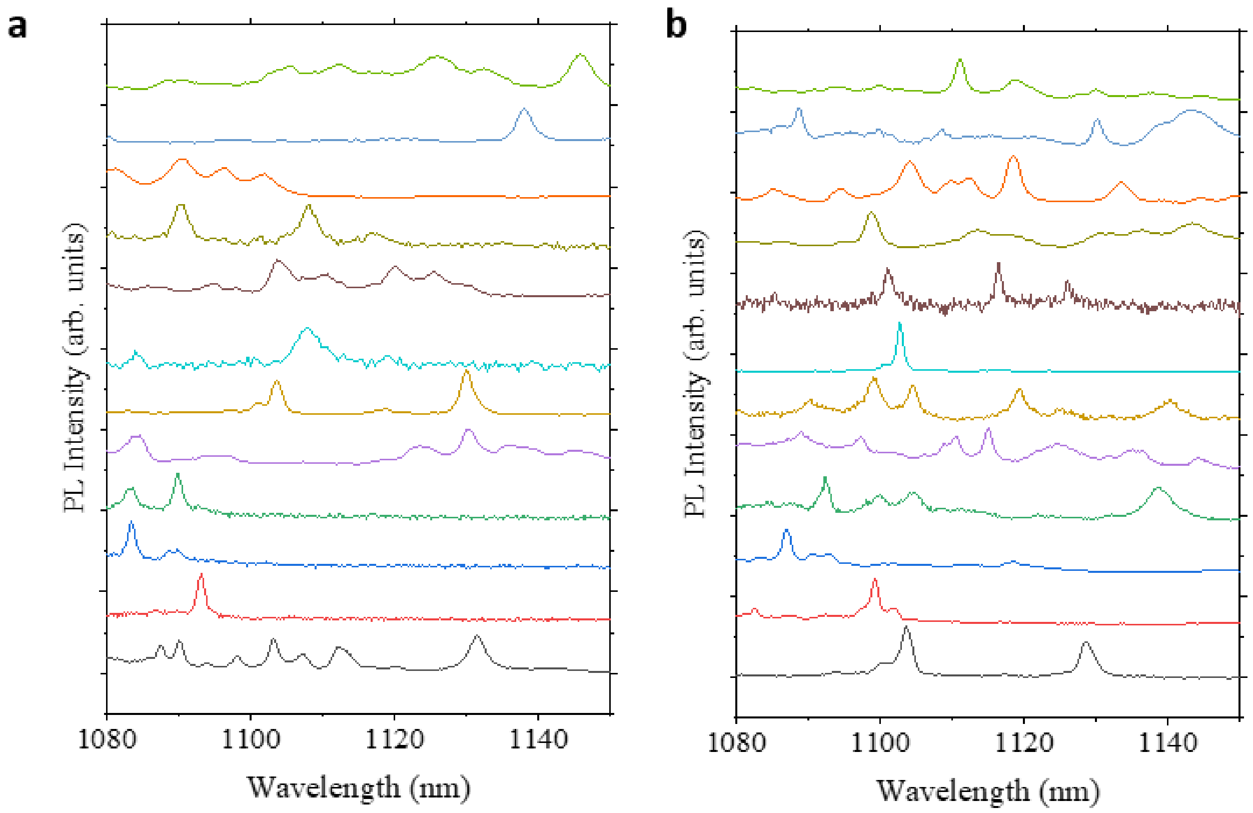

Supplementary Figure 4: The spectra of 24 localized $MoTe_2$ emitters obtained at 10–13 K.

Supplementary Note 3: Comparison of QE PL linewidth at 14 K and 4.9 K

Supplementary Figure 5 shows the PL spectra of a $MoTe_2$ QE measured at 14 K and 4.9 K temperatures showing linewidths (FWHM) of 0.87 meV and 0.6 meV, respectively. A blue shift of the PL emission energy is also observed at reduced temperature.

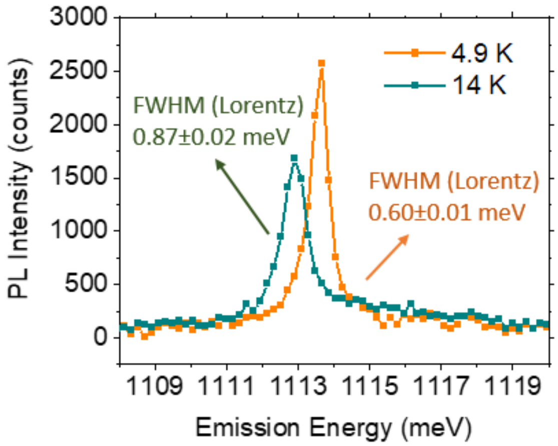

Supplementary Figure 5: The PL spectra of a QE measured at 14 K (teal) and 4.9 K (orange). The 4.9 K spectrum has a linewidth of 0.6 meV

Supplementary Note 4: Determining single photon detection efficiency and corrected emission rate of QEs

To measure the photon collection efficiency of our system, a tunable laser output at the wavelength of the QEs (1108 nm for QE of Figure 2 and 1540 nm for QE of Figure 3 in the main article) was coupled into the microscope. The signal reflected by a gold mirror passed through the PL collection path and the power was measured before the fiber coupler of the Quantum Opus superconducting nanowire single-photon detector (SNSPD) system, then compared with the laser power coupled into the objective to obtain the transmission efficiency value. This measurement yielded 5% and 4.4% transmission efficiency for 1108 and 1540 nm respectively. Then taking into account the collection efficiency of our 0.65 NA objective, the coupling efficiency to the SNSPD optical fiber (10% at 1108 and 30% at 1540 nm), and the SNSPD detector efficiency of 25% at 1108 nm and 85% at 1540 nm, we estimate the overall collection efficiency at 1108 nm and 1540 nm to be 0.035% and 0.31%, respectively. The overall collection efficiency at 1540 nm was an order of magnitude higher because the optical fiber and SNSPD detectors are optimized for 1550 nm.

Supplementary Figure 6 displays the SNSPD recorded photon counts of the QE presented in Figure 2 of the main article (1108 nm) under CW and pulsed excitation as well as the pulsed-laser excited

emission rate of the QE presented in main Figure 3 of the main article (1540 nm). The intensity fluctuation in all the figures are due to the drifting of the sample with respect to the laser focal spot. The data yield average count rates of 1.2 kHz (pulsed) and 9 kHz (CW) for the QE presented in Figure 2 of the main article, and 0.8 kHz for QE presented in Figure 3 of the main article. Normalizing these count rates with overall collection efficiencies estimated above leads to corrected emission rates given in the main article.

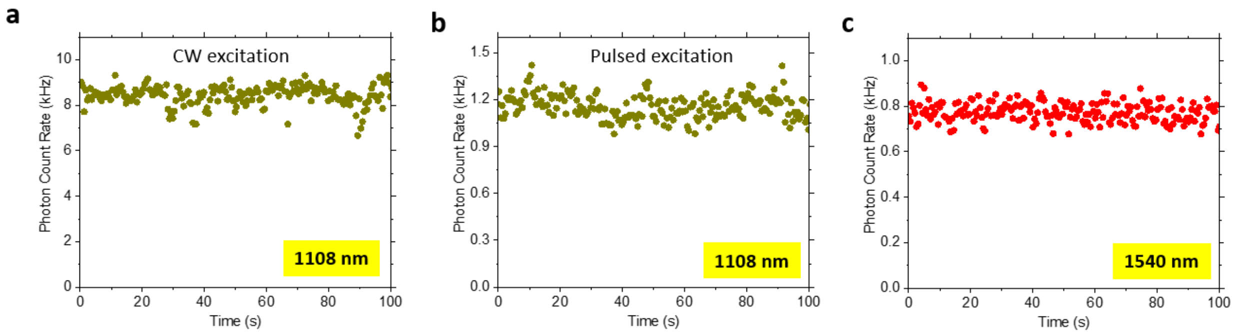

Supplementary Figure 6: Measured photon emission rate of QEs. a,b present the emission rate of the QE displayed in Figure 2 of the main article: **a,** under CW laser excitation; **b,** under 48.5 MHz pulsed laser excitation; **c,** the measured photon emission rate of the SPE displayed in Figure 3 of the main article under 303 kHz pulses excitation.

Supplementary Note 5: PL time trace of a near-band-edge emitter

Supplementary Figure 7 displays a PL (Supplementary Figure 7a) and PL time trace (Supplementary Figure 7b) of a localized emitter. The time resolution is 1.5 s and the spectral resolution is 0.4 meV. No blinking, photobleaching, or spectrum diffusion were observed.

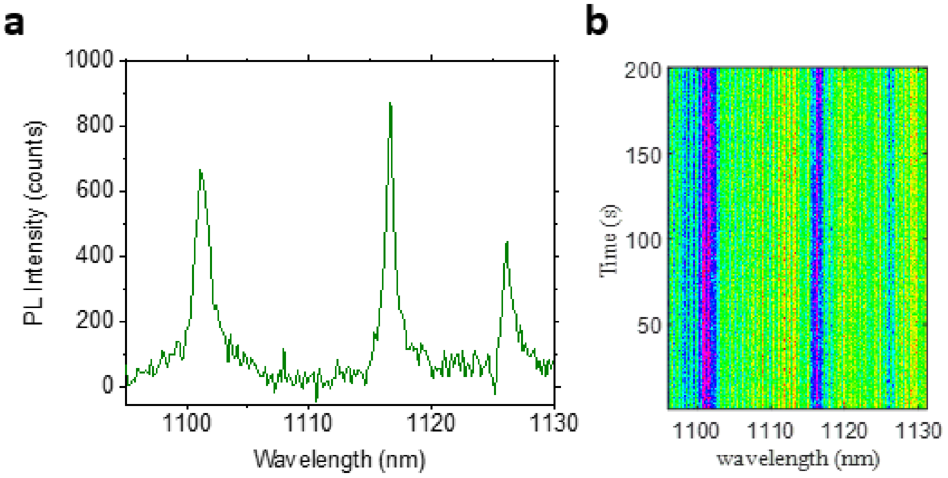

Supplementary Figure 7: Time-dependent PL of a QE. a, The PL spectrum of a localized emitter and **b**, its time dependence acquired over a 200 s integration time.

Supplementary Note 6: Near-band-edge emitter working at liquid-nitrogen temperature

Supplementary Figure 8 displays a near-band-edge SPE operating at 44 K and 77 K (liquid nitrogen temperature). At elevated temperatures, the PL intensity reduces and the lifetime also decreases, most likely due to thermally activated non-radiative recombination. Photon antibunching are clearly observed in both temperatures.

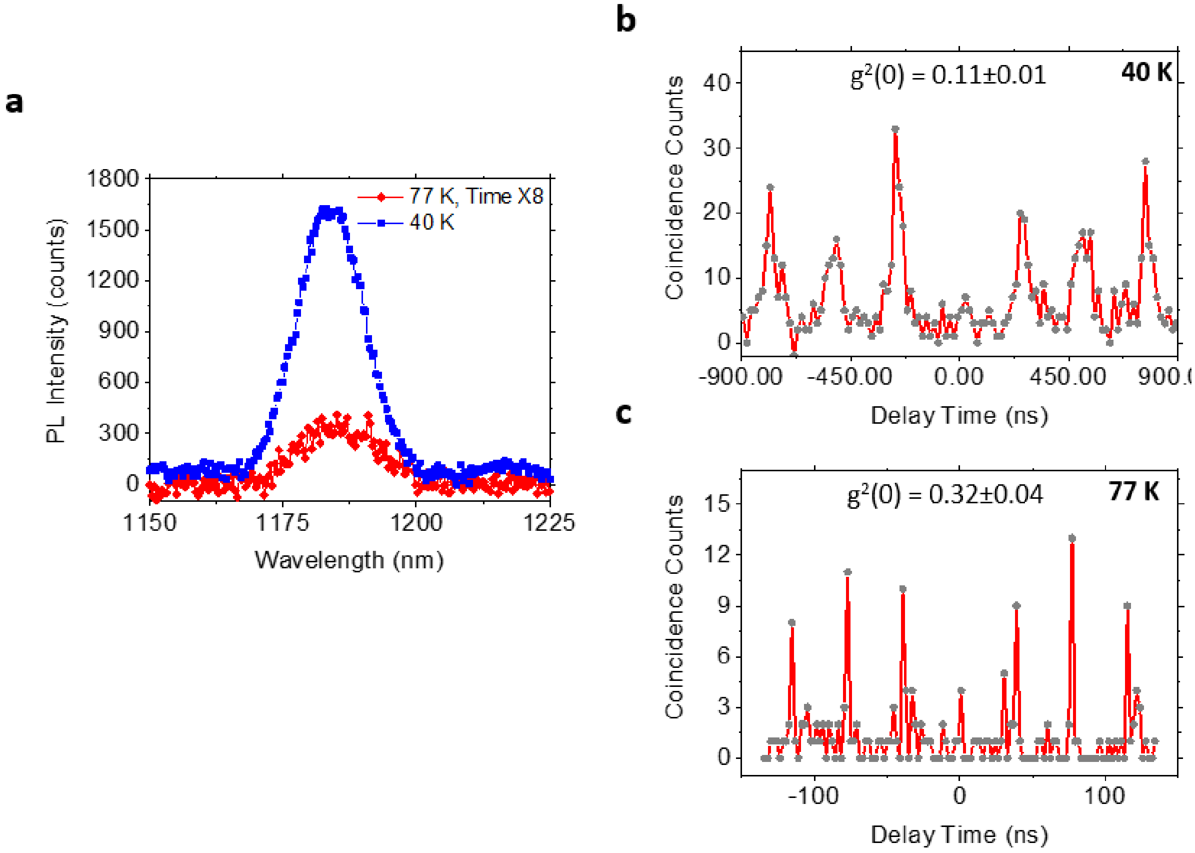

Supplementary Figure 8: $MoTe_2$ QE operated at liquid nitrogen temperature. a, PL spectra of a near-band-edge SPE measured at 40 K and 77 K. The integration time of the 77 K measurement is 8 times of that of the 40 K measurement indicating nearly 2 orders of magnitude decrease in emission rate. Pulsed $g^2(\tau)$ of the SPE measured at **b,** 40 K and **c,** 77 K clearly show photon antibunching.

Supplementary Note 7: Highly red-shifted localized emissions

Here we plot the spectra of 24 localized emitters with emission wavelength over 1150 nm (Supplementary Figure 9a,b**).** Most of the telecom emitters are found in multilayer $MoTe_2$ (layer number > 2) samples. The $MoTe_2$ 2D exciton peaks are usually too weak to be detected, as

multilayer $MoTe_2$ is an indirect bandgap semiconductor. However, we have occasionally observed monolayer (featured by its 1050 nm exciton peak) and bilayer (featured by its 1060 nm exciton peak) samples with 1550 nm telecom peaks, see Supplementary Figure 9c,d.

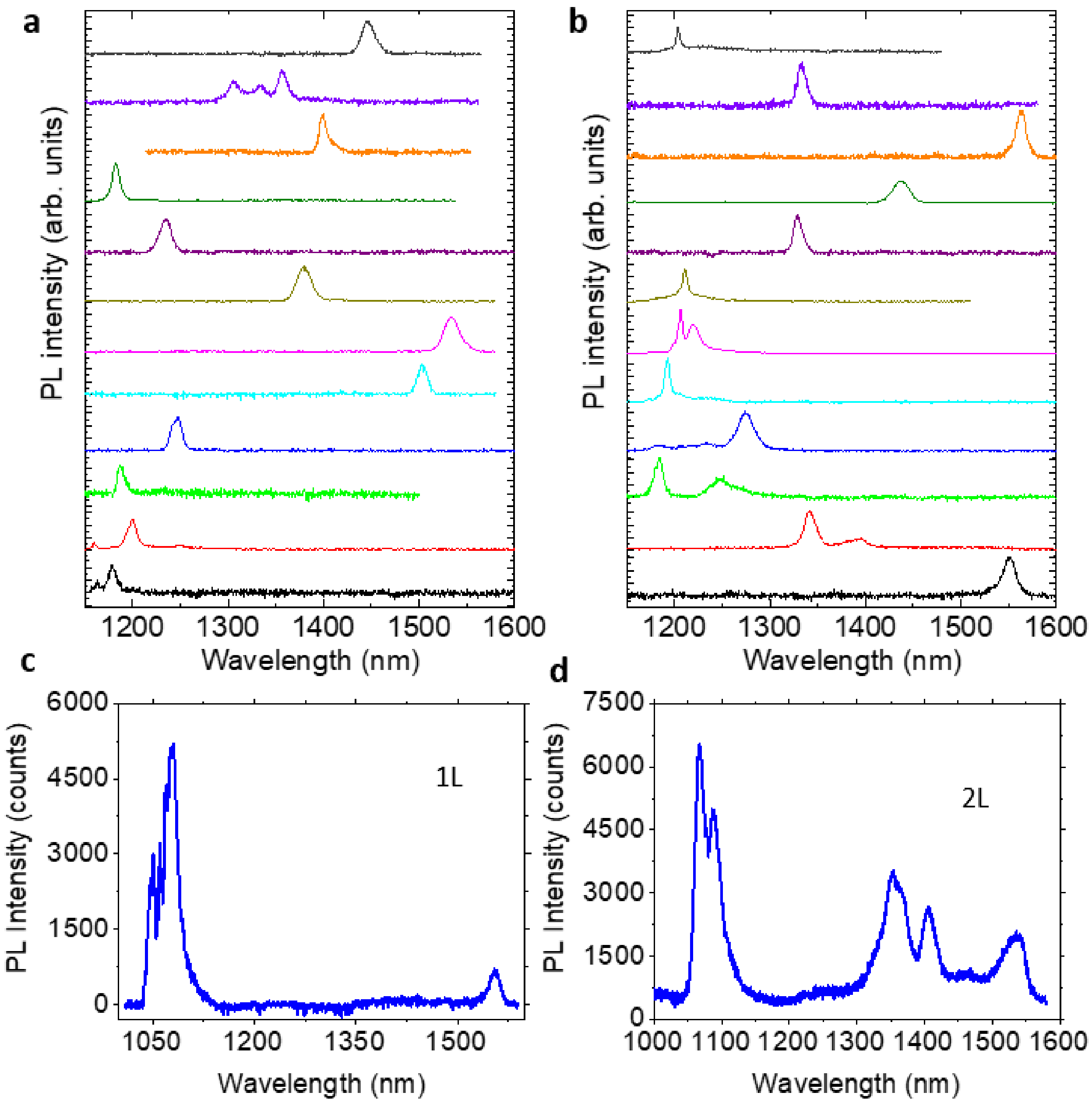

Supplementary Figure 9: PL spectra of telecom QEs. a,b, 24 localized emitters with emission wavelength above 1150 nm. **c**, A monolayer $MoTe_2$ localized emitter with a 1550 nm PL peak. **d**, A bilayer $MoTe_2$ localized emitter with a 1540 nm PL peak.

Supplementary Note 8: Nearly-unity emitter creation with high positioning accuracy

Supplementary Figure 10a shows a 5-6 layer $MoTe_2$ flake on a nano-pillar array. The layer thickness is identified by AFM. Supplementary Figure 10b presents the wide-field PL image of the flake with a 1300 nm long-pass filter applied in the collection path, showing near-unity creation of localized bright emitters. Supplementary Figure 10c is a PL spectrum of one of the emitters.

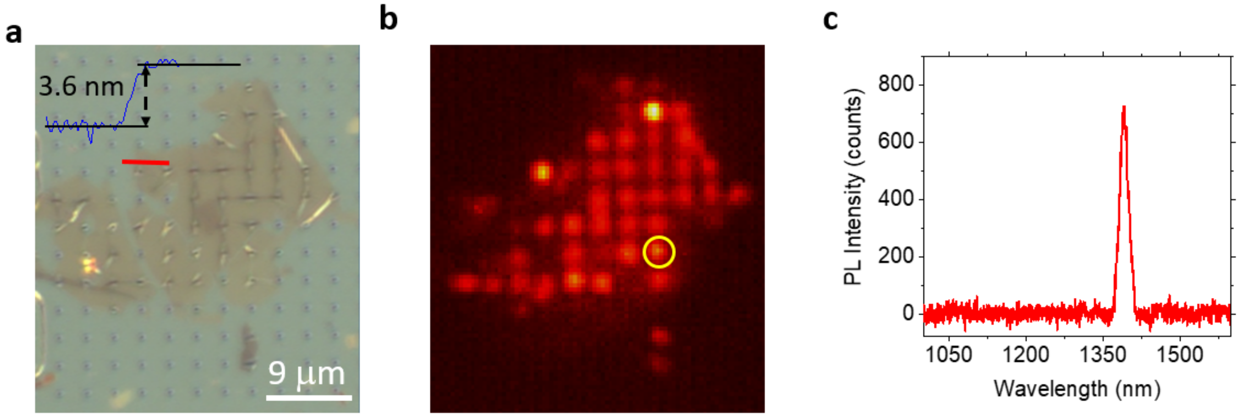

Supplementary Figure 10: a 5-6 layer MoTe2 flake on nanopillar array. a, Optical image (DIC mode) of an $MoTe_2$ flake on nano-pillar array. Inset: AFM cross section height profile taken at the red line region showing the sample is mainly a 5-6 layer flake; **b**, Wide-field PL image of the 5-6 layer $MoTe_2$ with a 1300 nm long-pass filter placed in the collection path; **c**, Representative PL spectrum obtained from the QE circled in yellow in panel **b**.

Supplementary Note 9: Second-order correlation data of SPEs emitting from 1.1 to 1.6 µm.

Supplementary Figure 11a shows 4 SPEs emitting from 1.1 to 1.6 µm. The second-order correlation data of each SPE are presented in Supplementary Figure 11b-e.

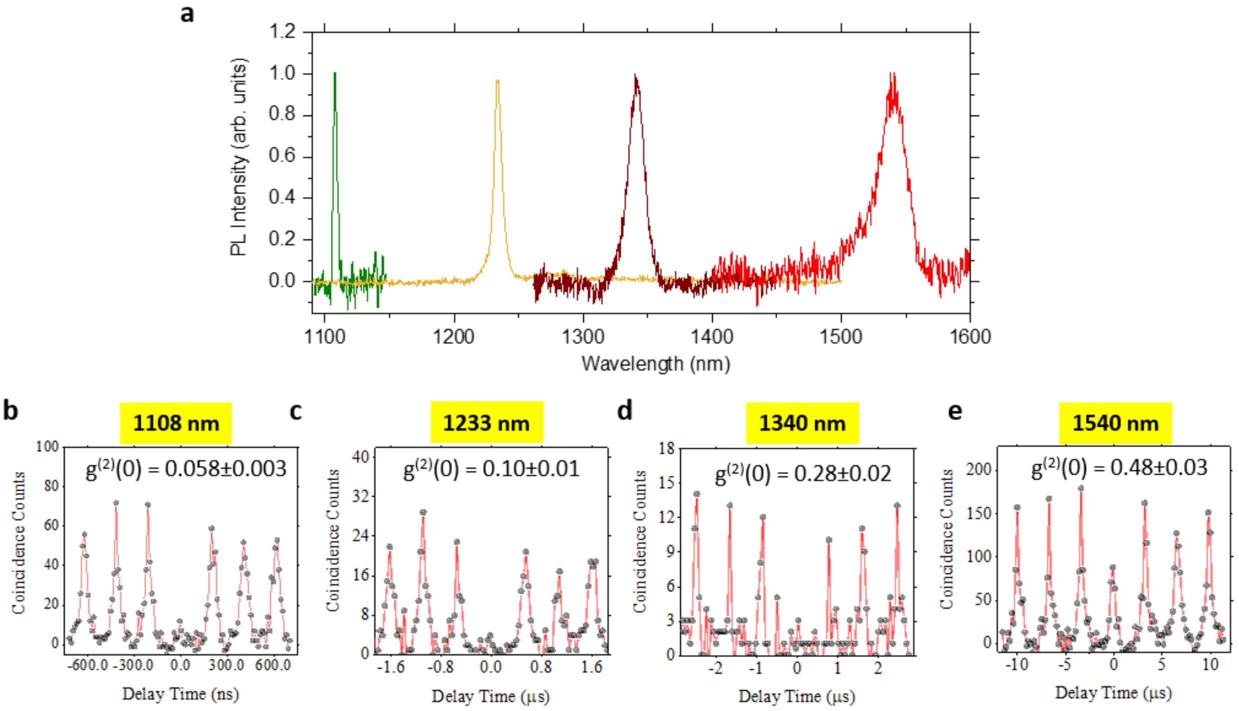

Supplementary Figure 11: Four QEs emitting from ~1100 to ~1540 nm. a, PL spectra of four localized emitters taken at 11 – 13 K temperature, with emission wavelength centered at 1108, 1233, 1340, and 1540 nm, respectively. **b-e,** The $g^2(\tau)$ data of the four QEs, respectively.

Supplementary Note 10: Correlating the PL spectra and TRPL decay curve of the QE shown in Figure 3b,c of the main article

The TRPL curve presented in Figure 3c of the main article and in Supplementary Figure 12a is fitted by a bi-exponential decay formula, $y = Ae^{-t/t1} + Be^{-t/t2} + C$. We obtained A = 69.6, B = 82.8, τ_1 = 163 ns and τ_2 = 1.13 μs. The ratio $A\tau_1 : B\tau_2$ = 1:8 is calculated to compare the ratio of the integrated PL intensity of two PL of peaks of Figure 3b. The PL intensity ratio of the 1510 nm shoulder peak and the 1540 nm telecom peak is calculated by integrating the PL counts from each fitted Gaussian peak shown in Supplementary Figure 12b. Because we applied a 1500 nm long-pass filter and a 1550±40 nm band-pass filter in the PL dynamics measurement, we only counted the “areas” with wavelength longer than 1510 nm, which is shadowed in yellow. From Supplementary Figure 12b we obtained a PL intensity ratio of 1:5. By comparing the PL intensity ratio obtained from the TRPL (1:8) and that obtained from the spectrum (1:5), we attribute the longer lifetime decay component to the brighter peak. As a result, the 1.13 μs decay component is assigned to the 1540 nm telecom peak.

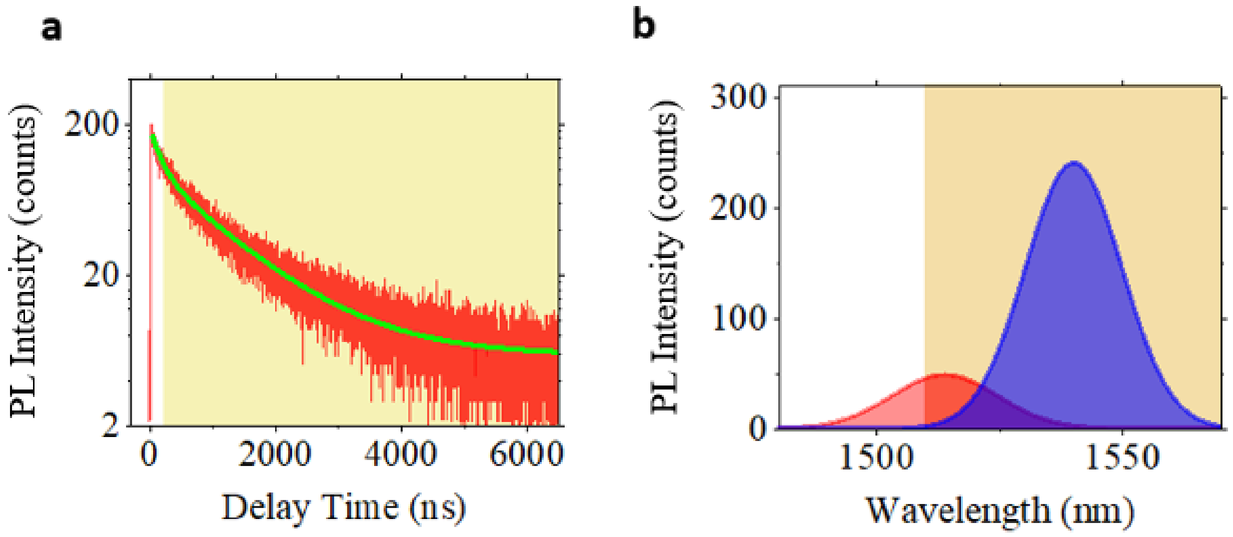

Supplementary Figure 12: Correlation the TRPL and PL curves of a telecom QE. a, The TRPL decay curve presented in Figure 3c (main article). The region shadowed in yellow is the time window utilized in photon correlation measurements; **b**, Gaussian fit curves presented in Figure 3b (main article). The "area" of each peak is denoted by the filled color. The region shadowed in orange is the spectrally filtered region used to obtain the TRPL data.

Supplementary Note 11: Photon correlation measurements and the time-gating technique

We performed both CW and pulsed laser photon correlation measurements using a two-channel SNSPD as the detector. The spectra were filtered either by a combination of long-pass and short-pass filters or a wavelength-tunable band-pass filter before entering the detector. Unfortunately, for the QE in Figure 3b-f of the main article, we do not have an appropriate optical filter to completely isolate the 1540 nm dominant peak from its shoulder peak at around 1510 nm, so the TRPL result in Figure 3c of the main article contains unwanted contributions from the small 1510 nm shoulder peak (Supplementary Figure 12b). To minimize the contribution from this short-lifetime shoulder peak in the pulsed autocorrelation measurements shown in Figure 3f of the main article, we applied a time gated 2nd order photon correlation approach to filter the photon in temporal domain after the measurement. Using the time tagged photons collected by a PicoQuant HydraHarp 400 photon counting system, we post-select the photons that are detected after certain time delay following the laser excitation (gate time) and reconstruct the 2nd order photon correlation trace from the selected photons. In our case, we have a relatively faster decay component with τ_1 = 163 ns and a slower decay component with τ_2 = 1.13 μs. By selecting the gate time to be 200ns (shadowed region in Supplementary Figure 12a denotes the time windows where photons are selected for the $g^{(2)}$ trace), 70% of the fast-decay contribution from the higher

energy could be removed along with 16% of the slow-decay, main spectral feature of interest. This choice yields $g^{(2)}(0)$ of 0.15 shown in Figure 3f. We note that we could increase the gate time to further reduce the contribution of the fast-decay component, but there is tradeoff as this also reduces the signal-to-noise ratio of the $g^{(2)}$ trace. Here 200 ns was found to be a good time gate value to balance the time-domain filtering effect and the signal quality. Although this time-gate technique is commonly used in quantum dots, it has not been demonstrated in 2D material research yet and thus we justify its use in detail in Supplementary Note 10-11.

Supplementary Note 12: Magnetic-field dependent valley Zeeman splitting

Supplementary Figure 13 plots the magnetic-field dependent valley Zeeman splitting of the localized emitter shown in **Figure 4a** of the main article, from which a *g*-factor of -3.61±0.02 is extracted.

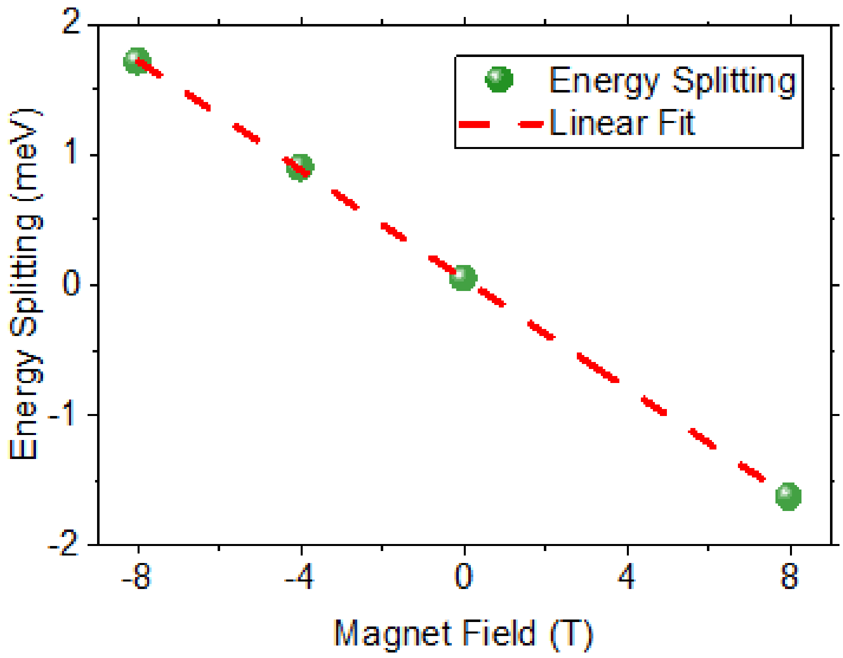

Supplementary Figure 13: Field-dependent valley Zeeman splitting of the emitter shown in Figure 4a of the main article. The linear fit is also presented.

Supplementary Note 13: A cross-linearly polarized doublet with an anomalously large zero field splitting

Although most of our cross-linearly polarized doublets show zero-field energy-splitting on the order of 1 meV, we occasionally observed anomalously large zero-field spitting for example in the SPE shown in Supplementary Figure 14a. The PL intensity of the lower energy peak is plotted as a function of linear polarization detection angle, shown in Supplementary Figure 14b. The data in Supplementary Figure 14b was found to be proportional to $\sin^2\theta$, showing the linearly polarized nature of the SPE.

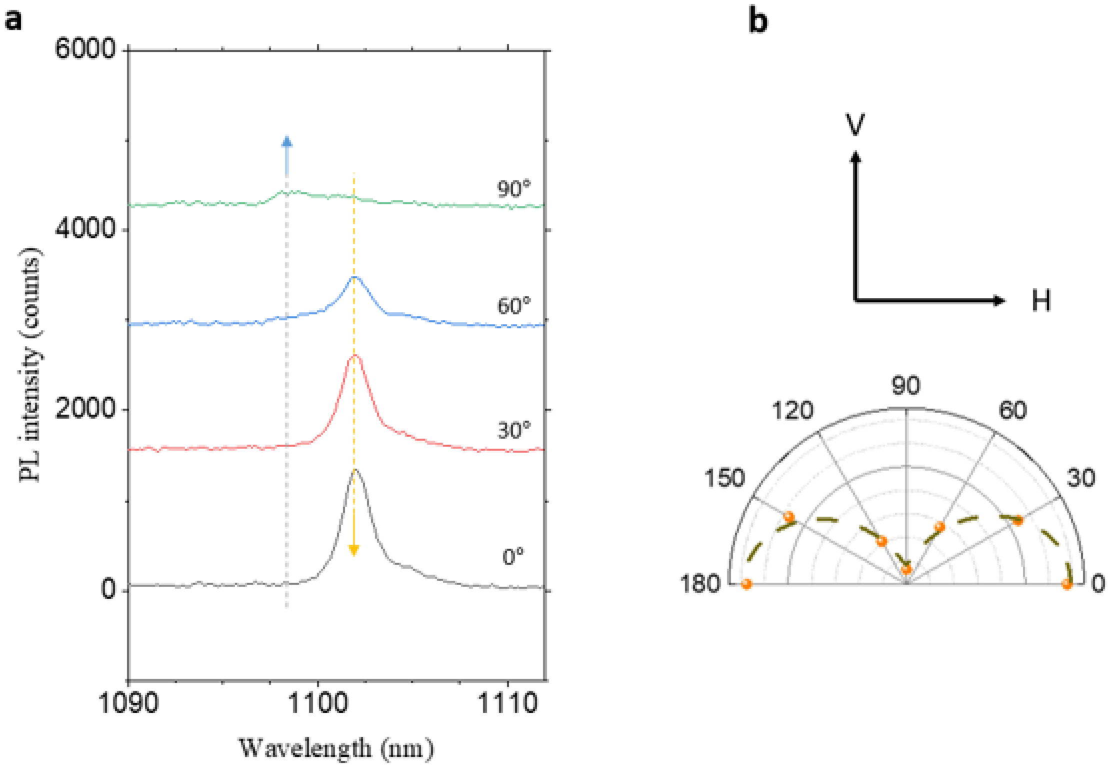

Supplementary Figure 14: Zero-order splitting and cross linear polarization of a QE. a, A quantum emitter with a giant zero-field splitting of $\Delta E_0 = 3.7$ meV; **b,** The polarization-dependent emission intensity and the sinusoidal $\sin^2\theta$ fit.

Supplementary Note 14: A diagram of the optical measurement setup

Supplementary Figure 15 shows a diagram of the optical path used in photon correlation and magneto-PL measurements.

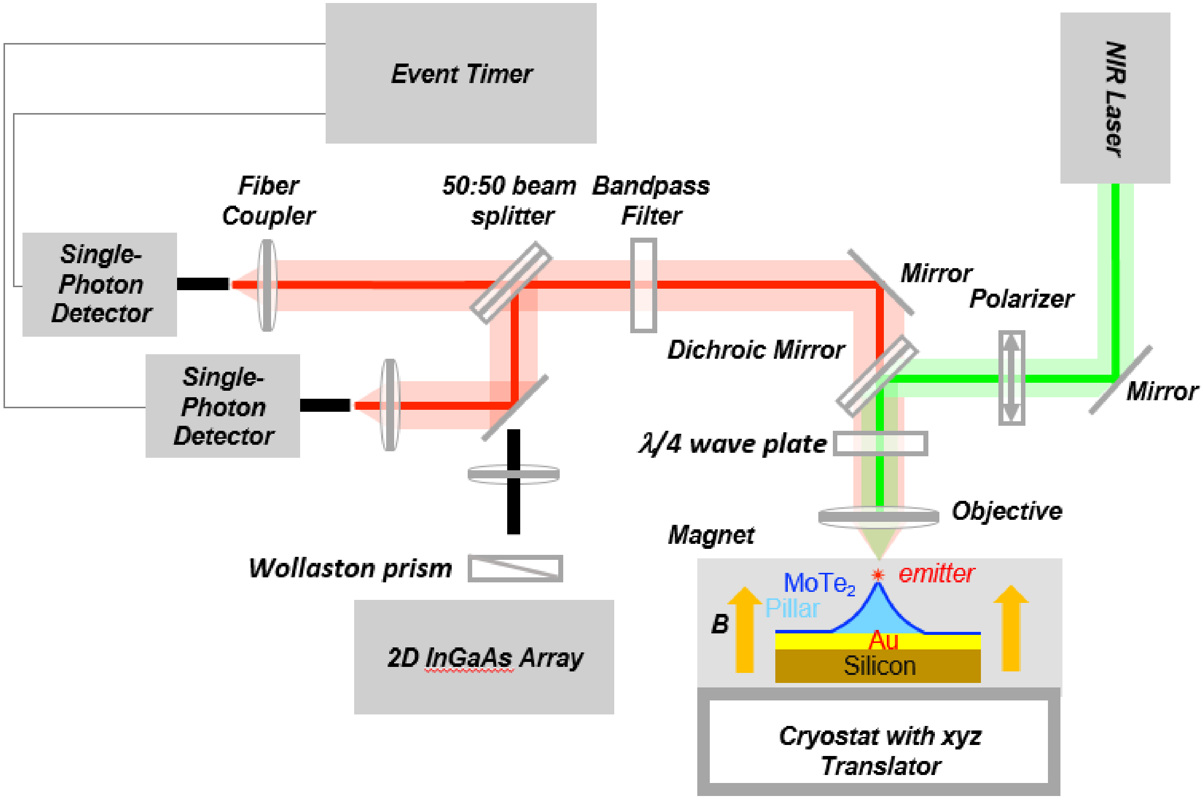

Supplementary Figure 15: An illustration of the optical measurement setup.
